# Supplementary material for: Splice-Junction-Based Mapping of Alternative Isoforms in the Human Proteome
Source: Cell Rep. Author manuscript; Available in PMC 2020 Jan 15. (PMC6961840; doi:10.1016/j.celrep.2019.11.026)
Supplement: 2 [file NIHMS1546469-supplement-2.zip › DF1/PXD010154/Prostate/Prostate_11_SZT2_GAPRPHLPVSAFSVDLSPR.pdf]

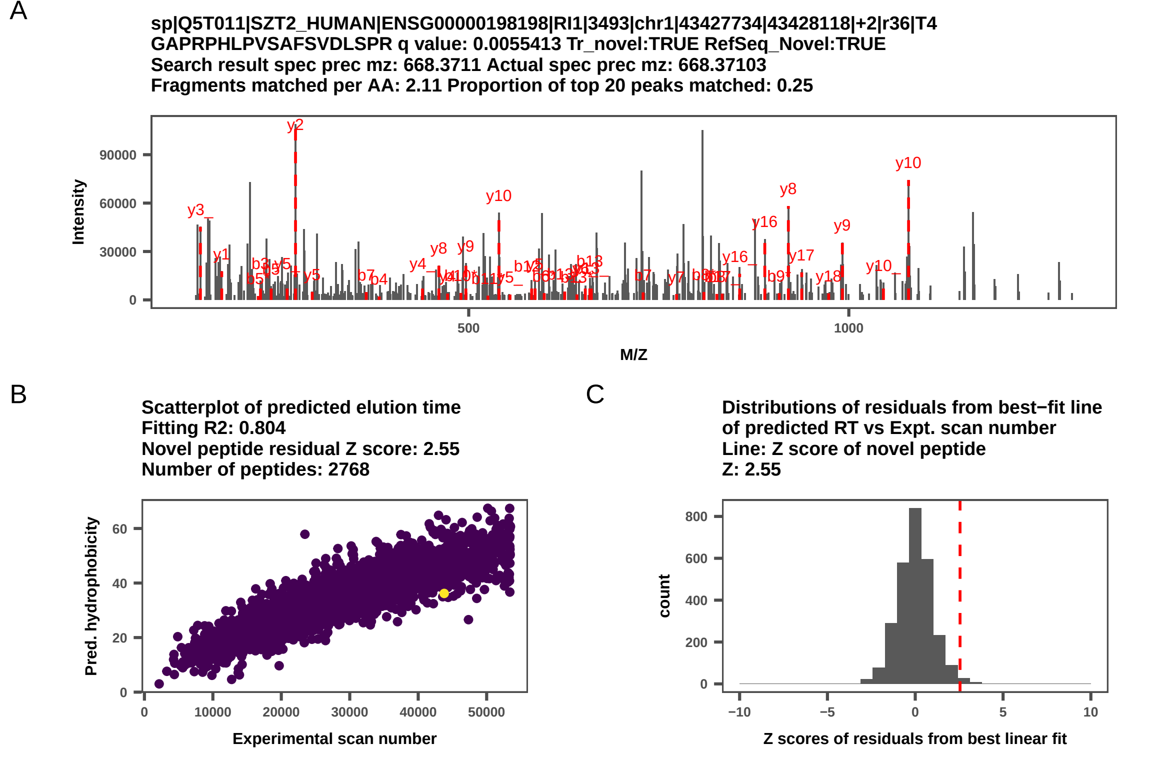
A
sp|Q5T011|SZT2_HUMAN|ENSG00000198198|RI1|3493|chr1|43427734|43428118|+2|r36|T4
GAPRPHLPVSAFSVDLSPR q value: 0.0055413 Tr_novel:TRUE RefSeq_Novel:TRUE
Search result spec prec mz: 668.3711 Actual spec prec mz: 668.37103
Fragments matched per AA: 2.11 Proportion of top 20 peaks matched: 0.25
Intensity
90000
60000
30000
0
M/Z
500
1000
y2
y10
y10
y8
y16
y9
y3
y1
y8
y9
y17
y18
B
Scatterplot of predicted elution time
Fitting R2: 0.804
Novel peptide residual Z score: 2.55
Number of peptides: 2768
Pred. hydrophobicity
60
40
20
0
0
10000
20000
30000
40000
50000
Experimental scan number
C
Distributions of residuals from best−fit line
of predicted RT vs Expt. scan number
Line: Z score of novel peptide
Z: 2.55
count
800
600
400
200
0
−10
−5
0
5
10
Z scores of residuals from best linear fit
